# ISG15 Promotes ERK1 ISGylation, CD8+ T Cell Activation and Suppresses Ovarian Cancer Progression

**DOI:** 10.3390/cancers10120464

**Published:** 2018-11-22

**Authors:** Tsz-Lun Yeung, Ching Chou Tsai, Cecilia S. Leung, Chi-Lam Au Yeung, Melissa S. Thompson, Karen H. Lu, Ralph S. Freedman, Michael J. Birrer, Kwong-Kwok Wong, Samuel C. Mok

**Affiliations:** 1 Department of Gynecologic Oncology and Reproductive Medicine, The University of Texas MD Anderson Cancer Center, Houston, TX 77030, USA; tyeung@mdanderson.org (T.-L.Y.); nick58tsai@gmail.com (C.C.T.); slleung@mdanderson.org (C.S.L.); CAu@mdanderson.org (C.-L.A.Y.); Melissa.Thompson@uth.tmc.edu (M.S.T.); khlu@mdanderson.org (K.H.L.); freedmanralphs@gmail.com (R.S.F.); kkwong@mdanderson.org (K.-K.W.); 2 Department of Obstetrics and Gynecology, Kaohsiung Chang Gung Memorial Hospital and Chang Gung University, Kaohsiung 83301, Taiwan; 3 Graduate Institute of Clinical Medicine, Kaohsiung Medical University, Kaohsiung 80708, Taiwan; 4 Comprehensive Cancer Center, Division of Hematology-Oncology, University of Alabama at Birmingham, Birmingham, AL 35294, USA; mbirrer@uab.edu

**Keywords:** ovarian cancer, ISG15, CD8+ lymphocyte, interferon, ISGylation

## Abstract

Increased number of tumor-infiltrating CD8+ lymphocytes is associated with improved survival in patients with advanced stage high grade serous ovarian cancer (HGSOC) but the underlying molecular mechanism has not been thoroughly explored. Using transcriptome profiling of microdissected HGSOC tissue with high and low CD8+ lymphocyte count and subsequent validation studies, we demonstrated that significantly increased ISG15 (Interferon-stimulated gene 15) expression in HGSOC was associated with high CD8+ lymphocyte count and with the improvement in median overall survival in both univariate and multivariate analyses. Further functional studies showed that endogenous and exogenous ISG15 suppressed ovarian cancer progression through ISGylation of ERK in HGSOC, and activation of NK cells and CD8+ T lymphocytes. These data suggest that the development of treatment strategies based on up-regulating ISG15 in ovarian cancer cells or increased circulating ISG15 in ovarian cancer patients is warranted.

## 1. Introduction

Approximately 22,000 new cases of epithelial ovarian cancer will be diagnosed in the United States each year. Over 16,000 deaths per year will occur, making this cancer the most lethal gynecologic malignancy [1]. Advanced high grade serous ovarian cancer (HGSOC), which accounts for the majority of new diagnoses, is notable for initial chemotherapy sensitivity using combination platinum and taxane chemotherapy following debulking surgery [2]. However, the vast majority of these women will have their cancer recur within 12 to 24 months after diagnosis and will die of progressively chemotherapy-resistant tumors [3,4]. The identification of prognostic or predictive markers for ovarian cancer is crucial for the development of therapeutic targets thus for the improvement of patient survival.

Recent studies demonstrated that intraepithelial T lymphocytes have an anti-tumor effect [5] and are associated with improved overall survival. They have been described in various solid tumors, including ovarian, endometrial, colon, and esophageal cancers [6,7,8,9,10]. In a study of 174 patients with advanced ovarian cancer, Zhang et al. noted improved survival rates in those with tumors with infiltrating CD3+ T lymphocytes [10]. Furthermore, Callahan et al. and Sato et al. showed that CD8+ cytotoxic T lymphocyte (CTL) infiltration in the ovarian tumor epithelium was associated with prolonged overall survival in patients with advanced HGSOC [9,11]. In spite of these studies, the mechanisms by which ovarian cancer cells modulate adaptive or innate immunity have not been thoroughly explored.

The present study aims to identify genes that are associated with increased intratumoral CD8+ lymphocyte density in advanced-stage HGSOCs. We further used both in vitro and in vivo models to determine the functional roles of one of the CD8+ lymphocyte density associated genes, ISG15 (Interferon-stimulated gene 15), which demonstrates significant differential expression between normal and malignant serous epithelia and is up-regulated by α and β interferons (IFNs) and has been shown to be associated with progression of ovarian cancer [12,13] and longer overall survival of ovarian cancer patients [14].

## 2. Results

### 2.1. Epithelial ISG15 Protein Expression Level is Associated with Intraepithelial CD8+ T Lymphocyte Density in HGSOC 

**Figure 1:**
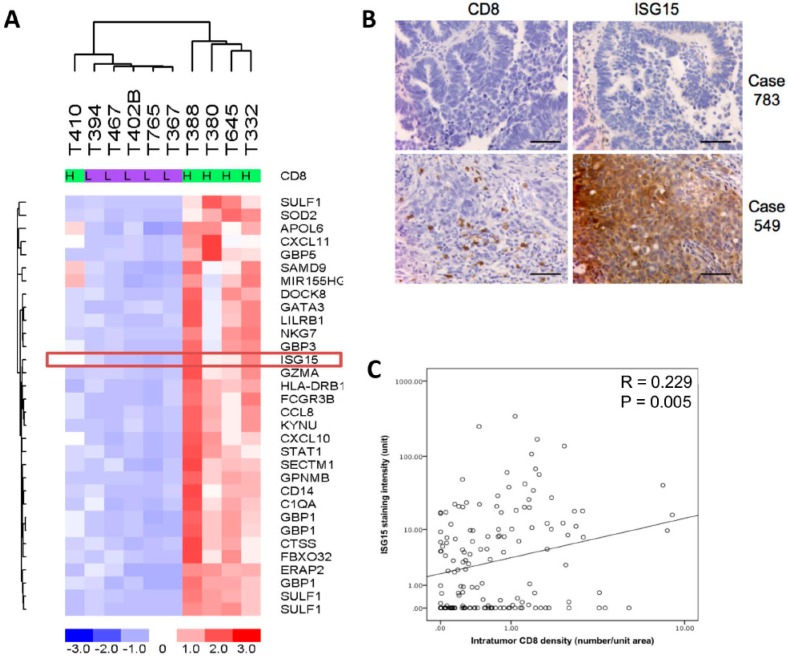
Interferon-stimulated gene 15 (ISG15) protein expression level is associated with intraepithelial CD8+ T lymphocyte density in high grade serous ovarian cancer (HGSOC). (**A**) Transcription profiling analysis on five microdissected HGSOC tissue samples with high density of intraepithelial CD8+ T lymphocyte and five samples with low density of intraepithelial CD8+ T lymphocyte showed that ISG15 expression levels is significantly higher in HGSOC samples with high intratumoral CD8+ cell densities when compared to those of the low intratumoral CD8+ cell densities. (**B**,**C**) Immunolocalization of ISG15 and CD8+ tumor infiltrating lymphocytes in 150 HGSOC samples showed a significant positive correlation between numbers of intratumoral CD8+ lymphocytes and ISG15 expression. (Bar = 100 μm).

Transcription profiling analysis on five microdissected HGSOC tissue samples with high densities of intratumoral CD8+ cells (>median +2 SD) and five samples with low densities of intratumoral CD8+ cells (<median −2 SD) revealed differentially expressed genes in patients with the high intratumoral CD8+ cell densities when compared to those with low densities (Figure 1A). ISG15, a gene whose expression has been shown to be up-regulated by interferons produced by activated CD8+ T lymphocytes and NK cells [15] was selected for validation studies. Immunolocalization of ISG15 and CD8+ tumor infiltrating lymphocytes in 150 HGSOC samples showed a significant positive correlation between numbers of intratumoral CD8+ lymphocytes and ISG15 expression in the epithelial component of the tumor tissue (Figure 1B,C). 

### 2.2. Differential ISG15 Expression in Normal and Malignant Serous Epithelia

**Figure 2:**
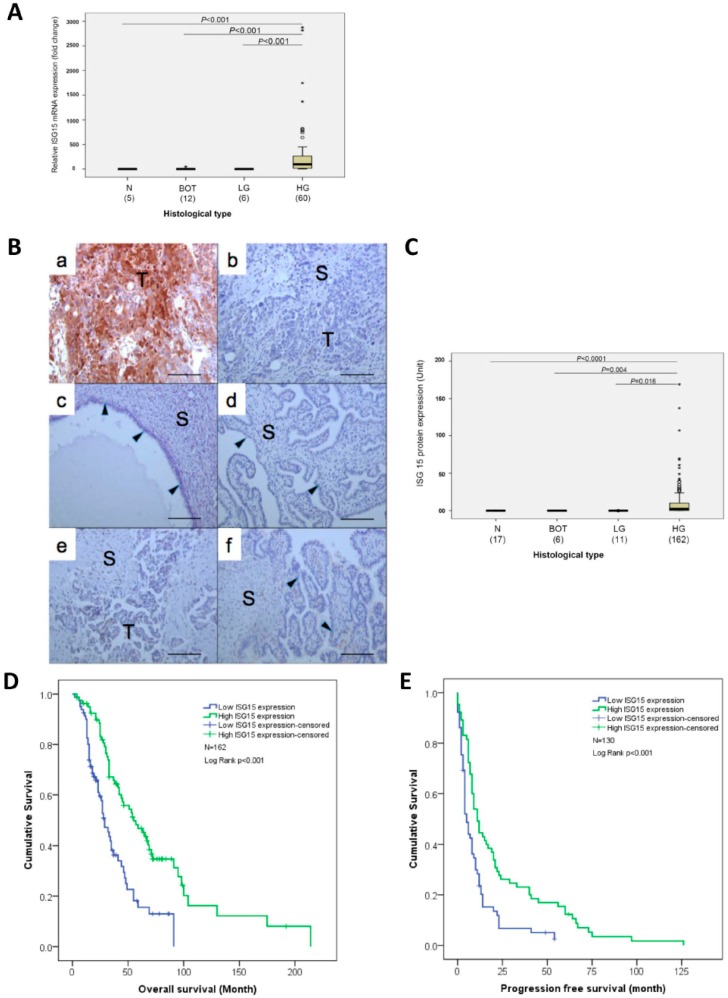
ISG15 protein expression in the tumor epithelium is associated with improved overall and progression-free survival. (**A**) Quantitative reverse transcriptase-polymerase chain reaction (qRT-PCR) analysis on RNA samples isolated from microdissected normal fallopian tube epithelia (N), serous borderline epithelia (BOT), low (LG) and high grade (HG) serous epithelia showed significantly higher levels of ISG15 expression in high grade serous epithelia than in other serous type epithelia. (**B**,**C**) Immunolocalization of the ISG15 protein in (**a**,**b**) high grade serous ovarian cancer, (**c**) normal ovarian cyst epithelium, (**d**) normal fallopian tubes, (**e**) low grade serous ovarian cancer and (**f**) borderline ovarian tumors, and showed high levels of ISG15 protein expression only in the epithelial component of the high grade serous ovarian cancer. (Bar = 100 μm). (**D**) Survival analysis using median ISG15 expression level as a cut off showed that HGSOC patients with high ISG15 expression in the tumor epithelium had a significantly longer median overall survival time (55 months) compared with patients with low expression of ISG15 (29 months) (*p* < 0.001). (**E**) Similarly, patients with high ISG15 expression in the tumor epithelium had a significantly longer median progression-free survival time (11 months) than in patients with low ISG15 expression (5 months) (*p* < 0.001).

To evaluate the differential expression of ISG15 mRNA in normal and malignant serous epithelia, qRT-PCR analysis was performed on RNA samples isolated from microdissected normal fallopian tube epithelia, serous borderline epithelia, low and high grade serous epithelia. The results showed significantly higher levels of ISG15 expression in high grade serous epithelia than in other serous type epithelia (Figure 2A). Immunolocalization of the ISG15 protein in normal fallopian tubes, normal ovarian surface epithelium, borderline ovarian tumors, and low and high grade serous ovarian cancer also showed high levels of ISG15 protein expression only in the epithelial component of the high grade serous ovarian cancer but not in the epithelial and stromal components of the normal fallopian tubes and other serous ovarian tumor samples (Figure 2B,C). 

### 2.3. ISG15 Protein Expression in the Tumor Epithelium is Associated with Improved Overall and Progression-Free Survival

Survival analysis using median ISG15 expression level as a cutoff on 162 previously untreated patients with advanced HGSOC showed that patients with high ISG15 expression in the tumor epithelium had a significantly longer median overall survival time (55 months, N = 81, 95% CI:36.9–73.1 months) compared with patients with low expression of ISG15 (29 months, N = 81, 95% CI:23.0–35.0 months), (*p* < 0.001), (Figure 2D). In addition, Kaplan–Meier analysis on 130 patients with progression-free survival data available, showed that patients with high ISG15 expression in the tumor epithelium had a significantly longer median progression-free survival time (11 months, 95% CI:7.8–14.2 months) than in patients with low ISG15 expression (5 months, 95% CI:3.5–6.5 months), (*p* < 0.001, Figure 2E).

In a proportional hazards, multivariate analysis with cytoreduction status and age adopted as covariates, low ISG15 expression by cancer cells was associated with a poorer overall survival (HR 2.265, 95% CI:1.5–3.43, *p* < 0.001), and progression-free survival (HR 1.909, 95% CI:1.28–2.84, *p* = 0.001) (Supplementary Table S1).

### 2.4. Endogenous ISG15 Expression Suppresses Ovarian Cancer Growth and Induces Apoptosis In Vitro

**Figure 3:**
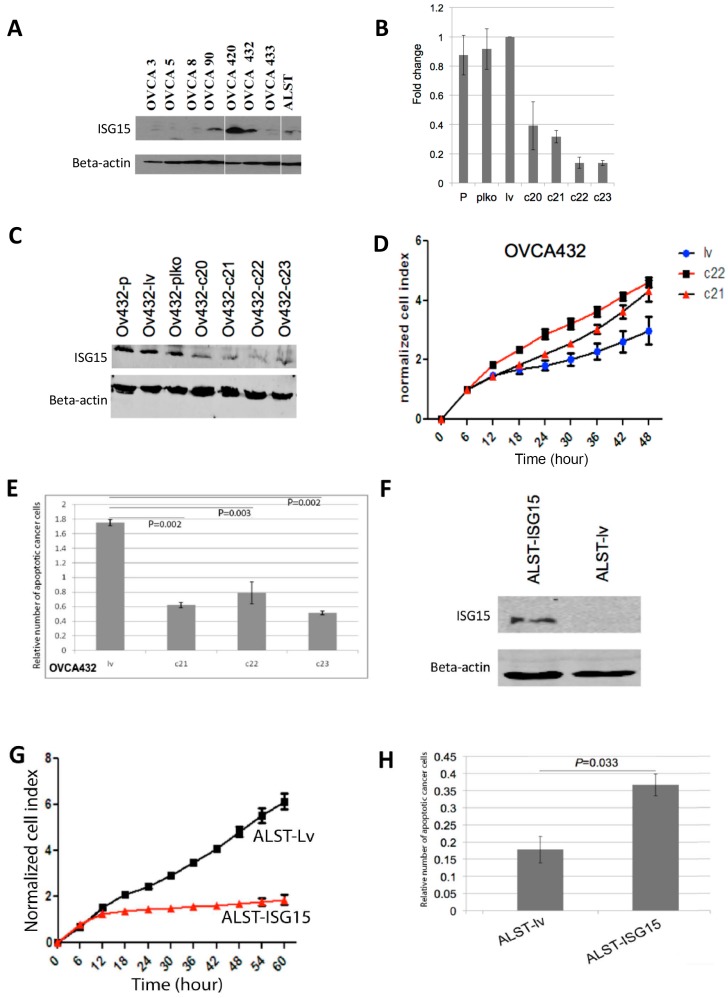
Endogenous ISG15 expression suppresses ovarian cancer growth and induces apoptosis in vitro. (**A**) Endogenous ISG15 expression levels were evaluated in eight ovarian cancer cell lines by Western bolting analysis. ISG15 expression in OVCA432 ovarian cancer cells was silenced by transduction of ISG15 specific shRNAs. ISG15 silencing was confirmed by (**B**) qRT-PCR and (**C**) Western blot analyses. (**D**) OVCA432 cells transduced with ISG15 shRNAs showed a significant increase in growth rates compared with those transduced with the control construct (*p* < 0.001). (**E**) Cell Death ELISA assay demonstrated a significant decrease in apoptosis in OVCA432 cells transduced with ISG15 shRNAs compared with the scramble sequence. (**F**) ALST ovarian cancer cells were transduced with a full-length ISG15 expression construct. Increased ISG15 protein expression in transduced cells were confirmed by Western blot analysis. In vitro experimental results showed a significant (**G**) decrease in growth rate and (**H**) increase in apoptosis in cells transduced with the ISG15 expression vector when compared with those transduced with the control vector.

To evaluate the effect of endogenous ISG15 on ovarian cancer growth in vitro, HGSOC cells OVCA432, which expressed high levels of endogenous ISG15 as confirmed by Western blot analysis (Figure 3A), were transduced with ISG15 specific shRNAs to evaluate the effect of ISG15 silencing on ovarian cancer growth. Successful ISG15 silencing was confirmed by both qRT-PCR and Western blot analyses on ISG15 mRNA and protein isolated from cells transduced with four different ISG15 specific shRNAs (c20, c21, c22, and c23), compared with those transduced with the vector alone (plko) or the vector with a scrambled shRNA sequence (lv) (Figure 3B,C). The results showed that OVCA432 cells with ISG15 expression silenced by transduction with c21 and c22 ISG15 shRNA showed a significant increase in growth rates compared with those transduced with the control construct lv (*p* < 0.001) (Figure 3D). In addition, Cell Death ELISA assay, which detects the presence of cell-free histone-complexed DNA fragments resulted from the induction of cell death/apoptosis, demonstrated a significant decrease in apoptosis in OVCA432 cells transduced with the three ISG15 shRNAs compared with the scramble sequence (Figure 3E). The effect of ISG15 silencing was further evaluated using another HGSOC cell line OVCA420, which also expressed high level of endogenous ISG15 and similar results were observed (Supplementary Figure S1A–C).

To evaluate whether high levels of endogenous ISG15 suppressed ovarian cancer growth and increased apoptosis, ALST ovarian cancer cells, which expressed low levels of ISG15 were transduced with a full length ISG15 expression construct. Increased ISG15 protein expression in transduced cells were confirmed by Western blot analysis (Figure 3F). Experimental results showed a significant decrease in growth rate and increase in apoptosis in cells transduced with the ISG15 expression vector when compared with those transduced with the control vector (*p* < 0.001) (Figure 3G,H).

### 2.5. Exogenous ISG15 Suppresses Ovarian Tumor Growth In Vitro

Since ISG15 has been shown to be secreted from monocytes and lymphocytes [16], we further evaluated whether ovarian cancer cells expressing high levels of endogenous ISG15 would secrete ISG15 protein into the extracellular space. In this experiment, four ovarian cancer cell lines (ALST, OV90, OVCA420, and OVCA432) expressing various levels of endogenous ISG15 were cultured in serum free media for 48 hours. Upon the completion of incubation, conditioned media were collected, concentrated and evaluated for the presence of ISG15 by Western blot analyses. Western blot results demonstrated a various amount of ISG15 in the conditioned media, which corresponded to the expression levels of ISG15 in the four ovarian cancer cell lines (Supplementary Figure S2A), suggesting that ISG15 is being actively secreted by the ovarian cancer cells into the extracellular space, cancer cell-derived ISG15 can engage in autocrine and paracrine signaling with different cell types in the ovarian tumor microenvironment.

To evaluate the effect of exogenous ISG15 on ovarian cancer growth, ALST cells, which secreted lowest levels of endogenous ISG15, were treated with two different physiological concentrations of ISG15 (0.5 and 2.5 ng/mL). The results showed ISG15 treated ALST demonstrated a significant dosage dependent decrease in growth rate in the presence of exogenous ISG15 compared with that in PBS treated cells (*p* < 0.001) (Supplementary Figure S2B).

### 2.6. ISG15 Overexpression Suppresses Ovarian Cancer Growth in Orthotopic Mouse Models

**Figure 4:**
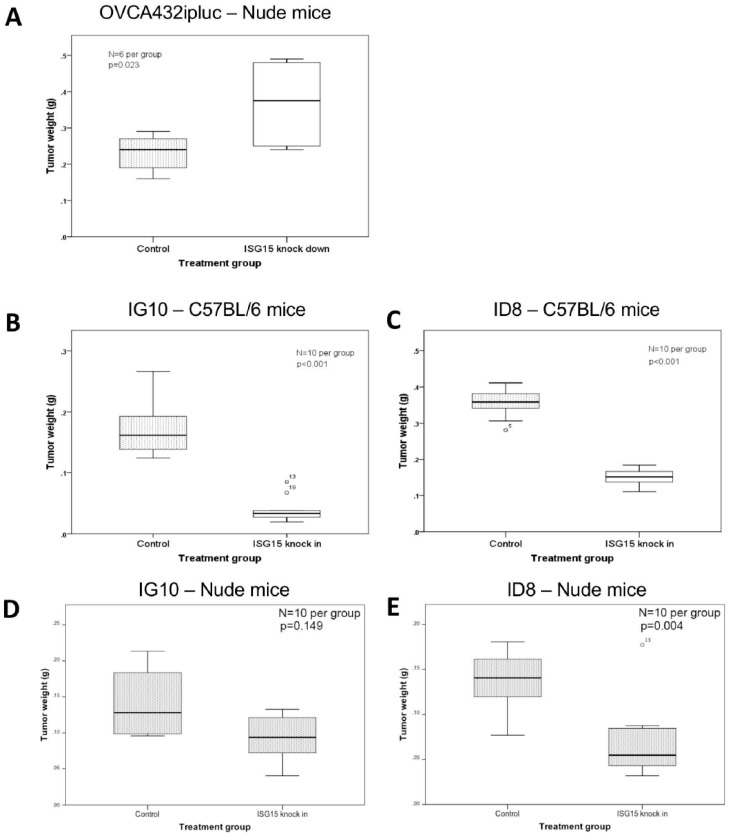
ISG15 overexpression suppresses ovarian cancer growth in orthotopic mouse models. (**A**) OVCA432 ovarian cancer cells were intraperitoneally injected into athymic nude mice. A significant increase in tumor burden was observed in mice injected with OVCA432 cells transduced with the ISG15 specific shRNA when compared with scrambles shRNA transduced control mice. To further evaluate the effect of ISG15 overexpression on ovarian cancer growth in an immune-competent microenvironment, mouse ovarian cancer cell lines (**B**) IG10 and (**C**) ID8 transduced with the full-length mouse ISG15 expression construct were intraperitoneally injected into immuno-competent C57BL/6 mice. Animal study results indicated a significant decrease in tumor weight for mice injected with cell lines transduced with the full length ISG15 expression construct when compared with those transduced with the control vector. To evaluate the effects of ISG15 expression on ovarian cancer progression in a T cell-depleted environment in vivo, both (**D**) IG10 and (**E**) ID8 cell lines were injected into C57BL/6 nude mice. The results showed a significant decrease in tumor size in mice injected with ISG15 transduced cancer cells when compared to those injected with the mock-transduced cells.

To study the effect of endogenous ISG15 on ovarian cancer growth in vivo, OVCA432 ovarian cancer cells with ISG15 expression silenced by shRNA were intraperitoneally injected into athymic nude mice. The results showed a significant increase in tumor burden in mice injected with OVCA432 cells transduced with the ISG15 specific shRNA c22 when compared with scrambles shRNA transduced control mice (*p* = 0.023) (Figure 4A). To further evaluate the effect of ISG15 overexpression on ovarian cancer growth in an immune-competent microenvironment, two mouse ovarian cancer cell lines (ID8 and IG10), which expressed low levels of endogenous ISG15, were transduced with the full-length mouse ISG15 expression construct followed by validation by qPCR (Supplementary Figure S3). Subsequently, both ISG15 transduced cell lines were intraperitoneally injected into immuno-competent C57BL/6 mice. Animal study results indicated a significant decrease in tumor weight for mice injected with cell lines transduced with the full length ISG15 expression construct when compared with those transduced with the control vector (*p* < 0.001) (Figure 4B,C), suggesting that ISG15 expression significantly suppressed ovarian tumor growth in the immune-competent in vivo model. To evaluate the effects of ISG15 expression on ovarian cancer progression in a T cell-depleted environment in vivo, both ID8 and IG10 cell lines transfected with the full-length mouse ISG15 and the control vector were injected into C57BL/6 nude mice. The results showed a significant decrease in tumor size in mice injected with ISG15 transduced cancer cells when compared to those injected with the mock-transduced cells (Figure 4D,E). In addition, the ISG15-mediated decrease in tumor weight was less profound in athymic C57 nude mouse model when compared to the immuno-competent C57BL/6 mouse model, suggested that the difference in the immune landscape, particularly the absence of T lymphocytes in the C57 nude mouse model, modulated the tumor suppressive effects of ISG15. 

### 2.7. ISG15 Induced ISGylation of ERK1 and Suppressed ERK1 Activity

We demonstrated that over-expression of ISG15 is associated with improved ovarian cancer patient survival, and overexpression of ISG15 suppressed ovarian cancer growth and increased ovarian cancer apoptosis. Since ISG15 modifies proteins through a process called ISGylation [17], which involves three cascade enzymes: An E1 enzyme (UBE1L), an E2 enzyme (UbcH8), and one of several E3 ligases to catalyze ISG15 conjugation of a specific protein, we hypothesized that increased ISGylation of proteins targeted by ISG15 in cells may mediate the suppressor effect of ISG15 in ovarian cancer progression. Proteomic studies using cells treated with IFN, immunoprecipitation and high-throughput immunoblotting have identified more than 300 cellular proteins that are potential targets for ISGylation. [17,18,19]. To identify ISGylated proteins that may play a role in suppressing ovarian cancer cell growth and invasion potential and inducing apoptosis, we performed pathway analyses on the cellular proteins that are potential targets for ISGylation. We identified a total of 39 candidate proteins that have been shown to be associated with cell proliferation, apoptosis, and invasion potential (Supplementary Table S2). Among them, ERK1 was selected for further studies since it has been implicated to be involved in cell motility and invasion potential, apoptosis and cell proliferation. ISGylation of ERK may therefore inactivate ERK and mediate the suppressor effect of ISG15 on ovarian cancer progression. To test this hypothesis, transcriptome profiling, reverse phase protein array (RPPA), and Western blot analyses were performed on OVCA5 and ALST transfected with the full-length ISG15 cDNA and the mock transfectants. Using the transcriptome profiling data, IPA Upstream Regulator Analysis and the Upstream Regulator Analysis revealed activation z-scores of −2.581 (*p* = 0.002) and −2.529 (*p* < 0.001) for the association between ERK inactivation and down regulation of ERK related downstream genes and in ALST and OVCA5 cells, respectively. These genes include: (1) Early growth response 1 (EGR1), an ERK1/2 regulated transcription factor which is responsible to cell growth and a negative regulator of apoptosis [20,21,22]; (2) Intercellular adhesion molecule 1 (ICAM1) which can be regulated by ERK signaling and is responsible to cell adhesion [23]; (3) Jun b proto-oncogene (JUNB) which has been shown to be up-regulated upon ERK activation [24] and knock down of JUNB promoted apoptosis in cancer cells [25]; (4) Finkel-Biskis-Jinkins murine osteosarcoma viral oncogene homolog (FOS), another ERK target [26], which binds to JUN and forms the AP-1 transcription complex; (5) insulin-like growth factor 1 receptor (IGF1R) which has been shown to be up-regulated by ERK activation and involved in the proliferation and survival of cancer cells [27,28,29]; and (6) heat shock 70 kDa protein 5 (HSPA5) whose expression requires ERK activation [30]. HSP5A silencing has been shown to decrease cancer cell proliferation and increase apoptosis [31,32,33].

**Figure 5:**
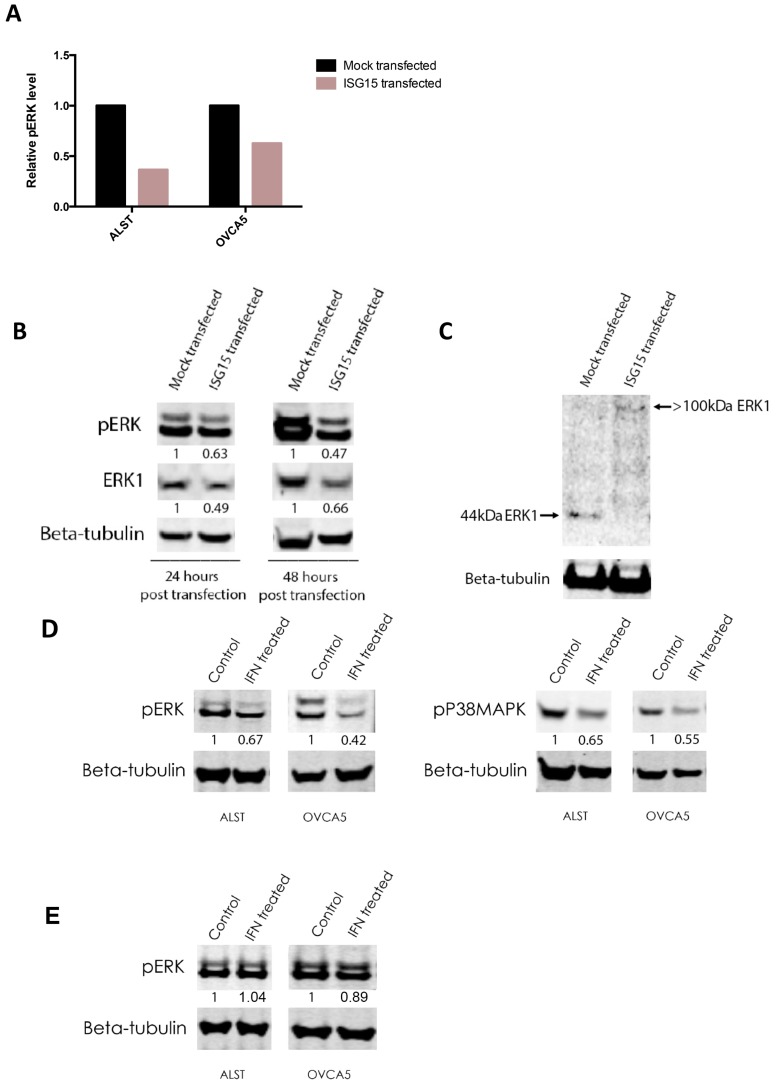
Endogenous ISG15 induced ISGylation of ERK1 and suppressed ERK1 activity. (**A**) Reverse phase protein array (RPPA) analysis showed that ALST and OVCA5 showed a 65% and 45%, respectively, decrease in the levels of Tyr204 phospho-p44 ERK1 when transfected with the full-length ISG15 expression construct when compared to mock. (**B**) Western blot analyses demonstrated a significant decrease in the levels of Tyr204 phospho-p44 ERK and total ERK1 expression in ISG15 transfected OVCA5 ovarian cancer cells when compared to mock transfected cells. (Numbers indicate band intensities relative to the corresponding control after normalization using intensities of housekeeping gene beta tubulin). (**C**) Western blot analysis detected the decrease in 44kDa ERK1 protein level and the presence of ISGylated ERK1 in ISG15 transfected OVCA5 cells. (**D**) Western blot analysis results showed that interferon gamma treated ALST and OVCA5 ovarian cancer cells had a significant decrease in phosphorylated ERK and P38MAPK protein levels when compared to control solvent treated cells. (Numbers indicate band intensities relative to the corresponding control after normalization using intensities of housekeeping gene beta tubulin). (**E**) Western blot analysis results showed that interferon gamma treatment on ISG15 silenced ALST and OVCA5 ovarian cancer cells had no effect on phosphorylated ERK protein levels when compared to control solvent treated cells. (Numbers indicate band intensities relative to the corresponding control after normalization using intensities of housekeeping gene beta tubulin).

Inactivation of ERK1 was further supported by RPPA and Western blot analyses. RPPA analysis demonstrated that ALST and OVCA5 showed a 65% and 45%, respectively, decrease in the levels of Tyr204 phospho-p44 ERK1 but not ERK2 when they were transfected with the full-length ISG15 expression construct when compared to mock transfectants (Figure 5A). Results of Western blot analyses demonstrated a significant decrease in the levels of Tyr204 phospho-p44 ERK expression in ISG15 transfected OVCA5 ovarian cancer cells when compared to mock transfected cells (Figure 5B). Furthermore, a decreased level of total ERK1 protein expression was observed in ISG15 transfected cancer cells. To evaluate whether the decrease in ERK1 level is a result of ISGylation, Western blot analysis was performed to detect the presence of ISGylated ERK1 in ISG15 transfected and mock transfected OVCA5 cells using an anti-ERK1 antibody. Experimental results showed that in addition to a decreased total ERK1 protein expression level, an increase in the intensity of the >100 kDa ISGylated ERK1 band was observed in the ISG15 transfectants when compared to the mock transfectants (Figure 5C). These findings further suggest that ISG15 suppresses ERK1 activity through ISGylation. Since ISG15 expression in ALST and OVCA5 cancer cells can be induced in the presence of interferon (Supplementary Figure S4), we further evaluated whether interferon treatment regulates ERK signaling via induction of ISG15 in ALST and OVCA5 ovarian cancer cells. Western blot analysis results showed that interferon gamma treated ALST and OVCA5 ovarian cancer cells had a significant decrease in phosphorylated ERK and P38MAPK protein levels when compared to control solvent treated cells (Figure 5D). Such inhibition in ERK signaling by interferon treatment was abrogated in ISG15 silenced cancer cells (Figure 5E), suggesting ISG mediated interferon-induced inhibition of ERK signaling.

### 2.8. ISG15 Activates CD8+ T Cells via NK Cell Activation

**Figure 6:**
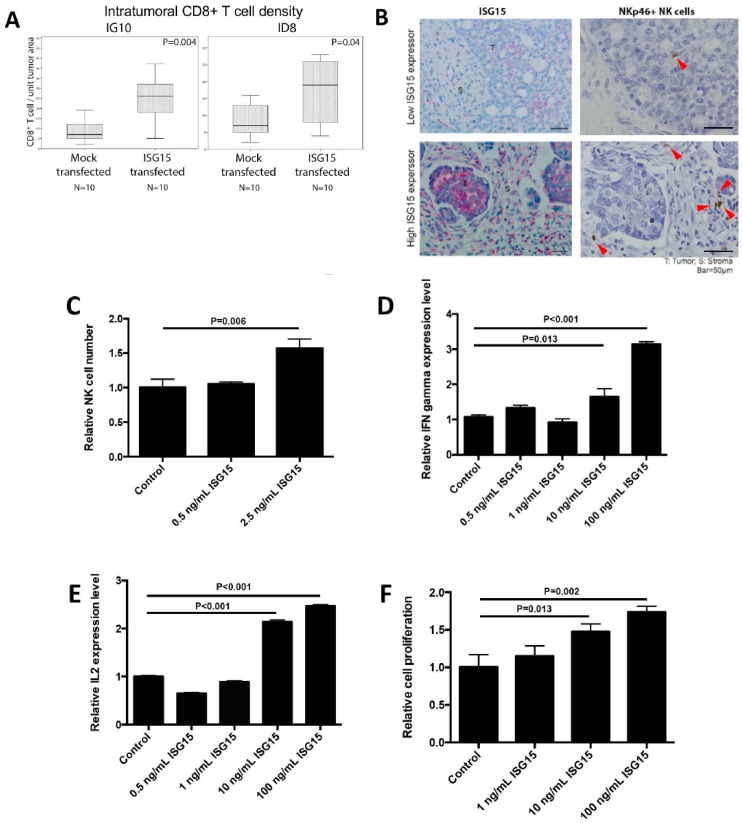
ISG15 activates CD8+ T cells via NK cell activation. (**A**) Immunolocalization of CD8+ T cells in tumor nodules isolated from C57BL/6 immune-competent mice injected with ID8 or IG10 cancer cells showed a significant increase in intraepithelial CD8+ T cells in tumors developed from mice injected with ID8 or IG10 transfected with ISG15 compared to those with the mock construct. (**B**) Immunostaining result showed a positive correlation between ISG15 expression in ovarian cancer cells and CD335 (NKp46) positive NK cell densities in ovarian tumor tissues (Bar = 50 μm). For the experiments performed to determine whether ISG15 could activate NK cells, experimental results showed that recombinant ISG15 significantly increased (**C**) NK cell proliferation, (**D**) IL-2 and (**E**) IFN-γ production in a dosage response manner. (**F**) To determine whether ISG15 treated NK cells could activate CD8+ T cells, conditioned media collected from NK cells treated with ISG15 were used to culture CD8+ T cells. CD8+ T cells treated with conditioned media from ISG15 treated NK cells showed a significant increase in cell number in a dosage response manner when compared to CD8+ T cells treated with conditioned media from NK cells treated with the control PBS.

We observed a significant correlation between increased ISG15 expression in ovarian cancer cells and increased intraepithelial CD8+ lymphocyte density in ovarian tumor tissue. To determine whether increased ISG15 expression in ovarian cancer cells led to increased CD8+ lymphocyte density in tumor tissue, immunolocalization of CD8+ cells was performed on tumor nodules isolated from C57BL/6 immune-competent mice injected with ID8 or IG10 transfected with ISG15 or the mock control. The results showed a significant increase in the numbers of intratumoral CD8+ lymphocytes in tumors developed from mice injected with ID8 or IG10 transfected with ISG15 compared to those with the mock construct (Figure 6A). We therefore hypothesized that high levels of free ISG15 secreted by tumor cells play a role in CD8+ T cell activation. We evaluated whether exogenous ISG15 directly affected CD8+ T cell activation. However, by treating CD8+ T cells with recombinant ISG15 protein we did not observe any effect of ISG15 on CD8+ T cell growth or on IL-2, IFN-γ, and granzyme B expression levels, suggesting that ISG15 has no direct effect on CD8+ T cell activation. Since NK cells have been shown to regulate CD8+ T cell response via cytokines secretion [34], we hypothesized that ISG15 might activate NK cells which secrete cytokines that subsequently activated CD8+ T cells. To test this hypothesis, we first determined whether there is any association between ISG15 expression in ovarian cancer cells and CD335 (NKp46) positive NK cell number in ovarian tumor tissues. The results showed a significant correlation between ISG15 expression levels in tumor cells and NK cell densities (Figure 6B). Next, we determined whether ISG15 could active NK cells. The results showed that recombinant ISG15 treatment significantly increased NK cell number, IL-2 production and IFN-γ production in a dosage response manner (Figure 6C–E). To determine whether ISG15 treated NK cells could activate CD8+ T cells, conditioned media collected from NK cells treated with ISG15 were used to culture CD8+ T cells. The results demonstrated that CD8+ T cells cultured with conditioned media from ISG15 treated NK cells showed a significant increase in cell number in a dosage response manner when compared to CD8+ T cells cultured with conditioned media from PBS solvent control treated NK cells (Figure 6F), suggesting that NK cells mediate the effect of ISG15 on CD8+ T cell activation.

### 2.9. Recombinant ISG15 Protein Suppressed Tumor Progression in Tumor-Bearing Animals

**Figure 7:**
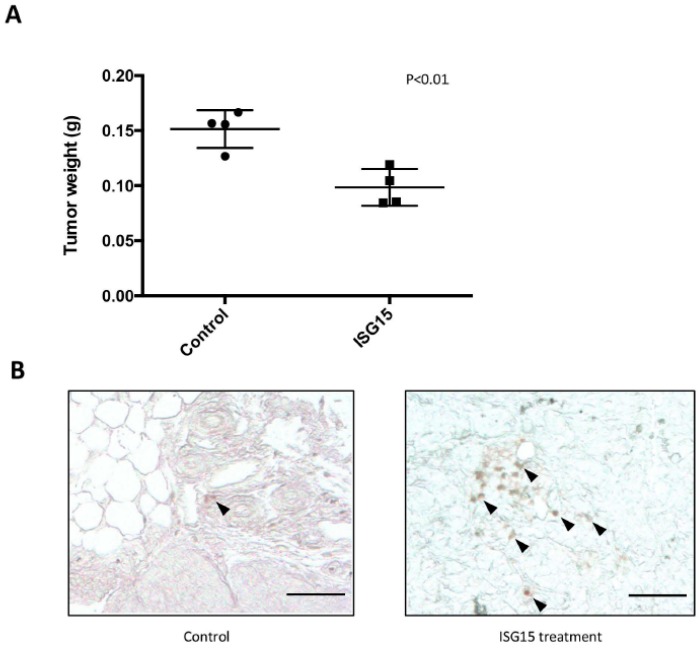
Recombinant ISG15 protein suppressed tumor progression in tumor-bearing animals. (**A**) Administration of 1 mg/kg recombinant ISG15 twice weekly suppress tumor progression in ID8 tumor-bearing C57BL/6 mice. A significant decrease in tumor weight in ISG15 treated mice was observed when compared to PBS treated mice. (**B**) Immunolocalization of CD8+ cells in tissue samples harvested from the ISG15 treated and the control mice demonstrated a significant increase in intraepithelial CD8+ lymphocyte density in ISG15 treated mice than in control mice. (Bar = 100 μm).

To determine whether administration of recombinant ISG15 could suppress tumor progression in mice, ID8 tumor-bearing C57BL/6 mice were treated with purified recombinant mouse ISG15 protein (1 mg/mL) through intraperitoneal injection. The results demonstrated a significant decrease in tumor weight in ISG15 treated mice when compared to those in PBS treated mice (*p* = 0.02) (Figure 7A). In addition, immunolocalization of CD8+ cells in omental tumor tissue samples harvested from the ISG15 treated and the control mice demonstrated a significant increase in intraepithelial CD8+ lymphocyte density in ISG15 treated mice than in control mice (Figure 7B) suggesting that ISG15 treatment facilitated T cell infiltration into the tumor tissue and promoted cancer cell eradication.

## 3. Discussion

Various immunological gene products in the pro-inflammatory tumor microenvironment support tumor growth and progression [35]. However, multiple lineages of immune cells are involved in antitumor responses. Among them, CD8+ NK cells have been shown to be able to kill tumor cells in various cancer models [36,37]. In addition, antigen-presenting cells, and dendritic cells in particular, process and present tumor derived antigens that activate CD8+ T cells via cross presentation [38]. Tumor-infiltrating CD8+ lymphocytes are associated with improved overall survival and have been described in multiple solid tumor types including ovarian cancer [10]. However, the genetic mechanisms underlying promotion or inhibition of CD8^+^ CTL infiltration in ovarian cancer are not fully understood. In this study, we identified significantly higher levels of ISG15 in samples with a high number of intratumoral CD8+ lymphocytes. The association is further confirmed in a separate validation set of specimens, which demonstrated that ISG15 protein expression levels in ovarian cancer cells indeed correlate with number of CD8+ tumor infiltrating lymphocytes. These data suggest that ISG15 overexpression in ovarian cancer cells may be associated with an increase in the number of CD8+ tumor infiltrating lymphocytes and ISG15 overexpression may also lead to improved patient survival. Both our Kaplan–Meier and Cox survival studies showed that high levels of ISG15 protein expressed by advanced high grade serous ovarian cancer cells are associated with improved patient overall survival. Darb-Esfanhani and colleagues also demonstrated through immunohistochemical analysis of ISG15 expression on 128 high grade serous ovarian cancer that patients with ISG15-positive tumors had a significantly longer overall survival than patients with ISG15-negative tumors. By correlation studies, they also showed that ISG15 expression was positively associated to expression of IκBα and phospho-IκBα, suggesting that ISG15 might be linked to the NFκB signaling pathway [14]. While both studies showed that ISG15 is a potential positive prognostic marker for ovarian cancer, the prognostic significance of ISG15 expression cannot be confirmed by analyzing the TCGA Agilent microarray dataset using cBioPortal [39]. The discrepancy may due to the fact that the TCGA dataset was generated from bulked tissue. Various numbers of immune cells such as lymphocytes, neutrophils, and monocytes present in the stromal, which have been shown to express ISG15 [16,40], will skew the ISG15 expression levels in the cancer cells and make the survival correlation insignificance. 

*ISG15* encodes a 15 kDa protein which is strongly induced in immune cells after type I IFN treatment or pathogen infection and has a significant sequence homology to ubiquitin [15]. ISG15 is composed of two ubiquitin-like domains, making it a linear dimer of an ubiquitin-like protein. High levels of ISG15 and its conjugates have been found in many types of primary tumors, including bladder, prostate, oral, and breast cancers [41,42,43,44]. Thus far, little is known about the function of ISG15 in ovarian carcinogenesis. Our immunohistochemistry data showed significantly lower levels of ISG15 expression in normal, benign and low grade epithelial than in high grade serous epithelia suggesting that ISG15 may have oncogenic properties. However, clinical correlation studies showed that high levels of ISG15 expression in ovarian cancer cells indeed correlated with improved survival, and our in vitro and in vivo data showed that endogenous ISG15 suppressed ovarian cancer growth and increased apoptosis. The apparent discrepancy is likely due to the fact that HGSOCs have a more active pro-inflammatory microenvironment with immune cells producing high levels of pro-inflammatory cytokines that up-regulates ISG15 in ovarian cancer cells [15]. In the context of HGSOC epithelia, high levels of endogenous ISG15 expression in ovarian cancer cells indeed play a tumor suppressor role.

Our data showed that intracellular ISG15 modulated the aggressive phenotype of ovarian cancer cells through inactivation of ERK1 by increasing ERK1 ISGylation in ovarian cancer cells. ISG15 is composed of two ubiquitin-like domains, making it a linear dimer of an ubiquitin-like protein. Intracellular ISG15 modifies proteins in a similar manner to ubiquitination through a process called ISGylation [15], which involves an E1 enzyme (UBE1L), an E2 enzyme (UbcH8), and one of the E3 ligases to catalyze ISG15 conjugation of a specific protein. These enzymes are induced by type I IFNs or other stimuli, such as exposure to viruses and lipopolysaccharide [15]. Recent studies identified over 300 cellular proteins, including ERK1, that are potential targets for ISGylation [17,18,19]. Jeon and associated showed that ISG15 played a role in the down-regulation of ΔNp63α in human breast cancer cells, suggesting that ISGylation may play a role in suppressing oncogenesis [17]. In addition, they also demonstrated that ISG15 modification of filamin B negatively regulated the JNK signaling pathway [45]. We showed here by Western blot analysis that up-regulated endogenous ISG15 in cancer cells increased the amount of ISGylated ERK1 and decreased the expression levels of phospho-ERK1 and ERK down-stream genes that are associated with tumor progression. These results confirmed that ERK1 is a target of ISG15. However, other ISG15 targets besides ERK1 may also be involved in suppressing ovarian cancer progression.

ISG15 is a known intracellular molecule involved in ISGylation and can be secreted by neutrophils, monocytes, and lymphocytes [40]. Recent studies have shown that secreted ISG15 can act on T and NK cells to induce IFNγ production and have suggested that ISG15 may play important roles in innate immunity [40]. Additionally, extracellular ISG15 acts as an immune adjuvant to enhance antigen-specific CD8+ T cell tumor immunity [46]. These data support our findings that exogenous ISG15 activated NK cells, which produce increased levels of cytokines such as IL2 and IFN-γ to stimulate CD8+ T cell proliferation. Future studies to evaluate the effects of ISG15 on tumor growth and immune response by manipulating the presences of different immune cell types, including NK cells, in animal models could provide valuable insight into the roles of ISG15 in the immune tumor microenvironment. Recently research findings showed that decreased BRCA1 expression resulted in increased ISG15 expression and protein ISGylation in human fallopian epithelial cells [47]. Follow up studies on the relationship between BRCA mutation status and ISG15 expression levels in ovarian cancer cells may help us to understand the underlying genetic components that drive ISG15 expression.

## 4. Materials and Methods

### 4.1. Patient Samples

Paraffin-embedded tissue from 162 high grade, International Federation of Gynecology and Obstetrics stage IIIB-IV (advanced-stage), 17 normal fallopian tubes, 6 serous borderline and 11 serous low grade serous ovarian tumor samples; and snap frozen tissue from 60 high grade serous ovarian cancer, 6 low grade serous ovarian cancer, 12 serous borderline ovarian tumors, and 5 normal fallopian tubes were used in this study. They were obtained from the ovarian cancer repository at The University of Texas MD Anderson Cancer Center. Tumor samples were collected from patients undergoing primary cytoreductive surgery for ovarian cancer. After surgery, patients received platinum-based combination chemotherapy. Optimal surgical cytoreduction was defined by a residual tumor no more than 1 cm in diameter. The overall survival duration was measured from the date of diagnosis to the date of death or censored at the date of the last follow-up examination. Clinical data, including age, cytoreduction status (optimal vs. suboptimal), and overall survival were abstracted from the records of the patients with HGSOC. Normal fallopian tube tissue samples were collected from patients who had undergone therapeutic salpingectomy for benign conditions, and were used as controls. All samples and clinical data were collected with the approval of the MD Anderson Institutional Review Board (the protocol number is LAB06-0412).

### 4.2. Cell Lines and Culture Conditions

Human ovarian cancer lines OVCA3, OVCA5, OVCAR8 and OVCA90 were obtained from the American Type Culture Collection (ATCC). OVCA420, OVCA432 and OVCA433 were a gift from Dr. Robert Bast’s laboratory at MD Anderson Cancer Center. The ALST cell line was developed at the Dana-Farber Cancer Institute. They were cultured in RPMI medium supplemented with 10% fetal bovine serum and 1% penicillin/streptomycin. Murine ovarian cancer cell lines ID8 and IG10 were cultured in DMEM medium supplemented with 10% fetal bovine serum and 1% penicillin/streptomycin. The identity of cell lines was authenticated using a microsatellite panel profiling and PCR tests were performed to ascertain that cell lines were pathogen-free.

### 4.3. Immunolocalization of Tumor-Infiltrating CD8+ Lymphocytes

Immunolocalization of CD8+ T cells was performed on 150 paraffin and 38 frozen sections prepared from advanced-stage serous ovarian cancer tissues using a monoclonal mouse, anti-human CD8 antibody (clone C8/144B, 1:50 dilution, DAKO, Santa Clara, CA, USA). In brief, tissue sections (paraffin-embedded) were deparaffinized, dehydrated, and antigen retrieval performed in Target Retrieval Solution (DAKO) with the pressure cooker at 120 °C for 20 minutes. Alternatively, frozen sections were fixed in methanol for 10 minutes. After blocking, sections were incubated with the primary antibody (anti-CD8) at room temperature for 90 minutes, washed two times with 1× Tris-Buffered Saline (TBS) (Boston BioProducts, Boston, MA, USA) and incubated with Polymer-AP (DAKO) for 30 minutes. CD8+ signals were visualized using a Vector Blue AP Substrate Kit III (Vector Laboratories, Burlingame, CA, USA). Digital photomicrographs of 10 fields of each slide were taken at ×20 magnification. Quantitative measurement of CD8+ cell density was determined using the Image-Pro Plus software (version 5.1; MediaCybernetics, Rockville, MD, USA).

### 4.4. Microarray Analysis

Using a Leica LMD6500 laser microdissection microscope, epithelial tumor tissue components were isolated from HGSOC patient tissue samples as previously described [48]. Transcriptome data previously generated from 38 microdissected advanced-stage HGSOC cases were used [49]. Immunolocalization of CD8+ lymphocytes was performed on FFPE tissue sections prepared from the same set of samples. Digital photomicrographs of 10 fields of each sample were taken at ×20 magnification. Quantitative measurement of intratumoral CD8+ cell density was determined using the Image-Pro Plus software (version 5.1; MediaCybernetics). Among the 38 cases, transcriptome data from 5 samples with high intratumoral CD8+ cell densities (>median +2 SD), were used to compare with those from 5 samples with low intratumoral CD8+ cell densities (<median −2 SD). The non-parametric Mann−Whitney test was used to identify probe sets with signal intensities that were significantly different between the high and low CD8 groups.

### 4.5. Evaluation of ISG15 Expression by Immunohistochemistry

Immunolocalization of ISG15 was performed on 17 normal fallopian tube tissue samples, 6 serous borderline ovarian tumors, 11 low grade serous ovarian carcinomas, and 162 high grade serous ovarian carcinomas. Slides were stained with a commercially available anti-ISG15 antibody (1:250; Sigma, St. Louis, MO, USA). ISG15 protein expression was visualized by 3,3’-diaminobenzidine (DAB). Normal rabbit IgG applied to high grade serous carcinoma samples with high levels of ISG15 expression was used as a negative control. Digital photomicrographs of representative areas of each slide were taken at ×20 magnification. Quantitative ISG15 staining intensity was determined using the Image-Pro Plus software (version 5.1; MediaCybernetics) as previously described [50]. The ISG15 staining intensity score was calculated by dividing the sum of the intensity values in a tumor area of interest by the number of pixels. This score was used to stratify patients according to low and high ISG15 expression using the median score as the cutoff point. Overall and progression-free survival analyses using both Kaplan−Meier modeling (with log-rank significance testing) and a Cox proportional hazards model were performed to determine the effect of ISG15 expression levels on overall survival and risk of death. A *p* value <0.05 was considered significant.

### 4.6. Effects of ISG15 Silencing on Ovarian Cancer Cell Proliferation, and Apoptosis

Endogenous ISG15 expression was evaluated in 8 ovarian cancer cell lines (OVCA3, OVCA5, OVCAR8, OVCA90, OVCA420, OVCA432, OVCA433, and ALST) using Western blot analysis. ISG15 was knocked down in ovarian cancer cells OVCA432 and OVCA420 by using four validated commercially available ISG15-specific shRNAs (Hs_ISG15_C20, Hs_ISG15_C21, Hs_ISG15_C22 and Hs_ISG15_C23) in lentiviral vectors (Sigma). Both a non-target scrambled shRNA sequence (lv) and the empty vector plko were used as negative controls (Sigma). Infection of ovarian cancer cells was performed in serum free medium. Validation of successful ISG15 knockdown was performed at the protein and mRNA levels using Western blot analysis, and qRT-PCR analysis, respectively.

A real-time cell proliferation assay was performed using the xCELLigence system (Roche Applied Science, Indianapolis, IN, USA). Cells were plated at 10,000 cells per well in serum-free Opti-MEM (Life Technologies, Grand Island, NY, USA) and allowed to attach for 12 hours. A unit-less parameter termed the cell index was derived and used to represent the cell number based on the measured relative change in electrical impedance that occurred in the presence and absence of cells in the wells [51]. The cell index was normalized to the baseline reading at time point 0 following attachment and transfection. Cell proliferation as measured according to the cell index was observed for 3.5 days.

Apoptosis assays were performed with a HGSOC cell line OVCA432 harvested transduced with Hs_ISG15_C21 and Hs_ISG15_C22 or with the non-target scrambled shRNA sequence using the Cell Death Detection cell ELISA system (Roche Diagnostics, Indianapolis, IN, USA) which colorimetrically measured and quantified the abundance of cell-free nucleosomes in each cell culture well [52]. A total of 5000 shRNA transduced ovarian cancer cells were plated in each of the wells in a 96-well plates and ELISA assay was performed at 72 hours after cell seeding. Using cancer cells transfected with non-targeted scrambled shRNA as the baseline, the relative increase in cancer cell apoptosis was calculated.

### 4.7. Effects of ISG15 Overexpression on Ovarian Cancer Cell Proliferation and Apoptosis

To determine the effect of ISG15 overexpression on ovarian cancer cell proliferation, ISG15 was overexpressed in serous ovarian cancer cell lines ALST and OVCA5, which have low endogenous ISG15 expression levels. The full-length ISG15 gene was delivered into the cells via transient transduction using ISG15 lentiviral particles (GeneCopoeia, Rockville, MD, USA). Then, 48 hours after transduction, cells were harvested, and overexpression of ISG15 in transduced cancer cells was confirmed. A cell proliferation assay and apoptosis assay were performed on ISG15-overexpressing cells or control mock-transduced cells as described above.

### 4.8. Effect of Exogenous ISG15 on Ovarian Cancer Cell Proliferation

To evaluate the effect of exogenous ISG15 on ovarian cancer growth, Western blot was first performed on conditioned media collected from 4 ovarian cancer cell lines (ALST, OV90, OVCA420, and OVCA432) together with known concentrations of purified ISG15 (ProSpec, East Brunswick, NJ, USA). ALST cells that showed low levels of ISG15 secretion were treated with 0.5 and 2.5 ng/mL ISG15. Cell proliferation was evaluated by the real-time cell proliferation assay using the xCELLigence system (Roche Applied Science) as described above.

### 4.9. Effect of ISG15 Silencing and Overexpression on Ovarian Tumor Growth in Orthotopic Mouse Models

To evaluate the effect of ISG15 silencing on ovarian tumor growth in vivo, 2 × 10^6^ OVCA432 cells transduced with Hs_ISG15_C22 or with the non-target scrambled shRNA sequence were suspended in phosphate-buffered saline (PBS) were intraperitoneally injected into 6 nude mice. After 7 weeks, mice were euthanized by CO_2_ inhalation followed by cervical dislocation and submitted to necropsy. The mice and their tumors were weighed, and resected tumors were processed for histologic evaluation.

To evaluate the effect of ISG15 over-expression in ovarian cancer cells in immune-competent mice, 2 × 10^6^ mouse ovarian cancer cells ID8 and IG10, which expressed low-levels of ISG15, were stably transduced with mouse ISG15 in lentiviral vector or the control vector and intraperitoneally injected into 10 C57BL/6 mice. After 5 weeks, mice were euthanized by CO_2_ inhalation followed by cervical dislocation and submitted to necropsy. Mice and their tumors were weighed, and resected tumors were processed for histologic evaluation.

### 4.10. Effect of ISG15 Treatment on Ovarian Tumor Growth and CD8+ T Cell Densities in an Immune Competent Mouse Model

To evaluate the effect of ISG15 protein treatment silencing on ovarian tumor growth in vivo, 2 × 10^6^ IG10 mouse ovarian cancer cells were intraperitoneally injected into 10 nude mice. Two weeks after cancer cell injection, mice were randomized. Half of the animals were given 1 mg/kg of recombinant mouse ISG15 protein (R&D Systems, Minneapolis, MN, USA) via intraperitoneal injection twice for 6 weeks, while the remaining half were given intraperitoneal injection of sterile PBS solvent control throughout the course of the experiment. At the experimental endpoint, all animals were euthanized by CO_2_ inhalation followed by cervical dislocation and submitted to necropsy. Tumors were weighed, and resected tumors were processed for immunostaining of CD8+ T cells using a polyclonal rabbit anti-mouse CD8 antibody (LifeSpan BioSciences, Seattle, WA, USA).

### 4.11. Statistical Analysis

The product-limit method of Kaplan and Meier was used to estimate survival curves and the curves compared with the log-rank statistic. A multivariate Cox proportional hazards model was used to estimate hazard ratios among covariates. The Mann−Whitney *U* Test was used to compare medians of continuous variables between two independent samples. The Pearson correlation and Spearman rank correlation were utilized where appropriate to assess the linear relationship between two continuous variables. A logistic regression model was used to predict the outcome of a dichotomous variable (CD8 density) from one predictor variable (ISG15 expression). All statistical analyses were performed with IBM SPSS 21. A *p*-value <0.05 (two-sided test) was considered statistically significant.

## 5. Conclusions

We demonstrated that increased ovarian cancer cell-derived ISG15 production inhibited ovarian cancer growth by suppressing ERK activity through ISGylation in an autocrine manner. In addition, increased secretion of ISG15 by ovarian cancer cells activated NK cells via paracrine signaling, which subsequently increased CD8+ cell proliferation rates. These findings support our observation that increased ISG15 expression levels in ovarian cancer cells is associated with increased intraepithelial CD8+ T cell density and improved patient survival rates.

## Supplementary Materials

The following are available online at http://www.mdpi.com/2072-6694/10/12/464/s1, Figure S1: ISG15 silencing suppresses OVCA420 cell growth; Figure S2: Exogenous ISG15 suppresses ovarian tumor growth in vitro; Figure S3: ISG15 overexpression in mouse ovarian cancer cell lines; Figure S4: Interferon treatment on ovarian cancer cells upregulates ISG15 expression; Table S1: Multivariate survival analysis of ISG15 expression in ovarian cancer patients; Table S2: Target proteins of ISGylation.
