# Supplementary material for: ISG15 Promotes ERK1 ISGylation, CD8+ T Cell Activation and Suppresses Ovarian Cancer Progression
Source: Cancers (Basel). 2018 Nov 22;10(12):464. doi: 10.3390/cancers10120464 (PMC6316352; doi:10.3390/cancers10120464)
Supplement: Supplementary file 1 [file cancers-10-00464-s001.pdf]

cancers

MDPI

Supplementary Material

ISG15 Promotes ERK1 ISGylation, CD8+ T Cell Activation and Suppresses Ovarian Cancer Progression

Tsz-Lun Yeung, Ching Chou Tsai, Cecilia S. Leung, Chi-Lam Au Yeung, Melissa S. Thompson, Karen H. Lu, Ralph S. Freedman, Michael J. Birrer, Kwong-Kwok Wong and Samuel C. Mok

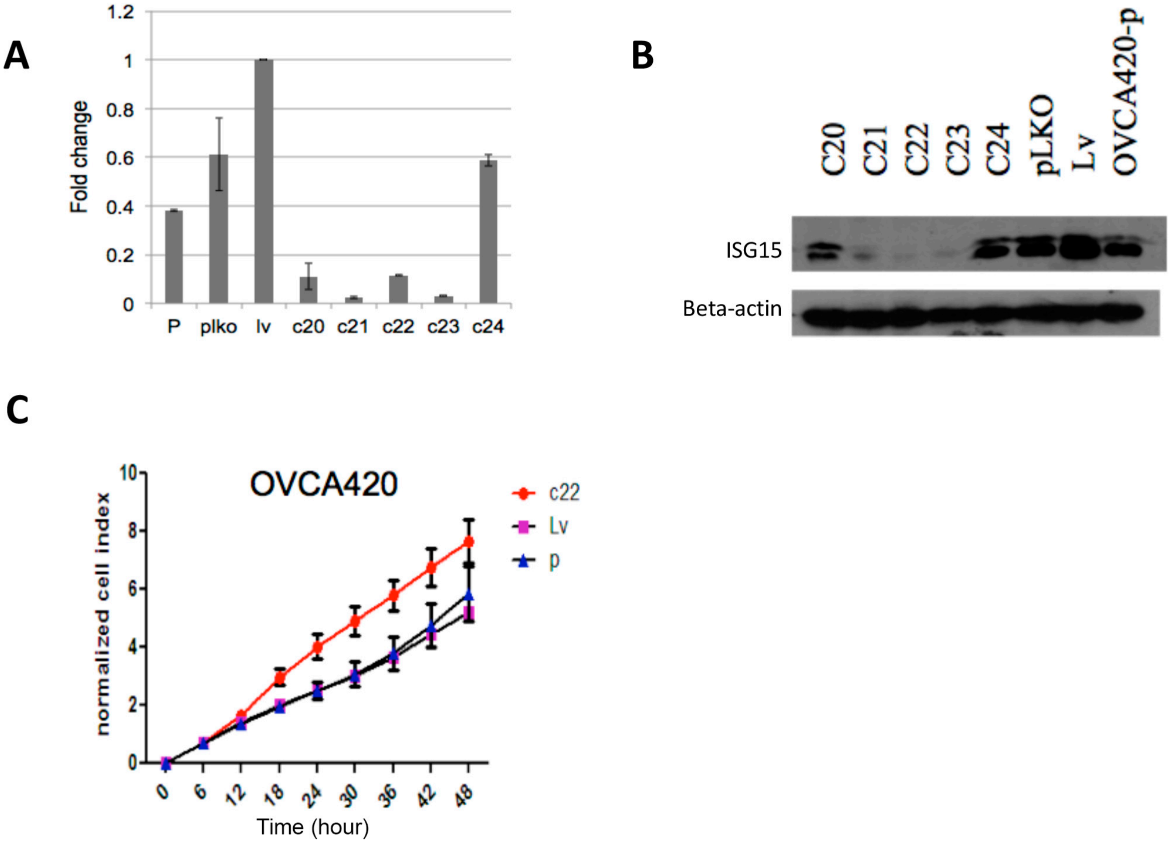

Figure S1. ISG15 silencing suppresses OVCA420 cell growth. ISG15 expression in OVCA420 ovarian cancer cells was silenced by transduction of ISG15 specific shRNAs. ISG15 silencing was confirmed by (**A**) qRT-PCR and (**B**) Western blot analyses. (**C**) OVCA432 cells transduced with ISG15 shRNAs showed a significant increase in growth rates compared with those transduced with the control construct.

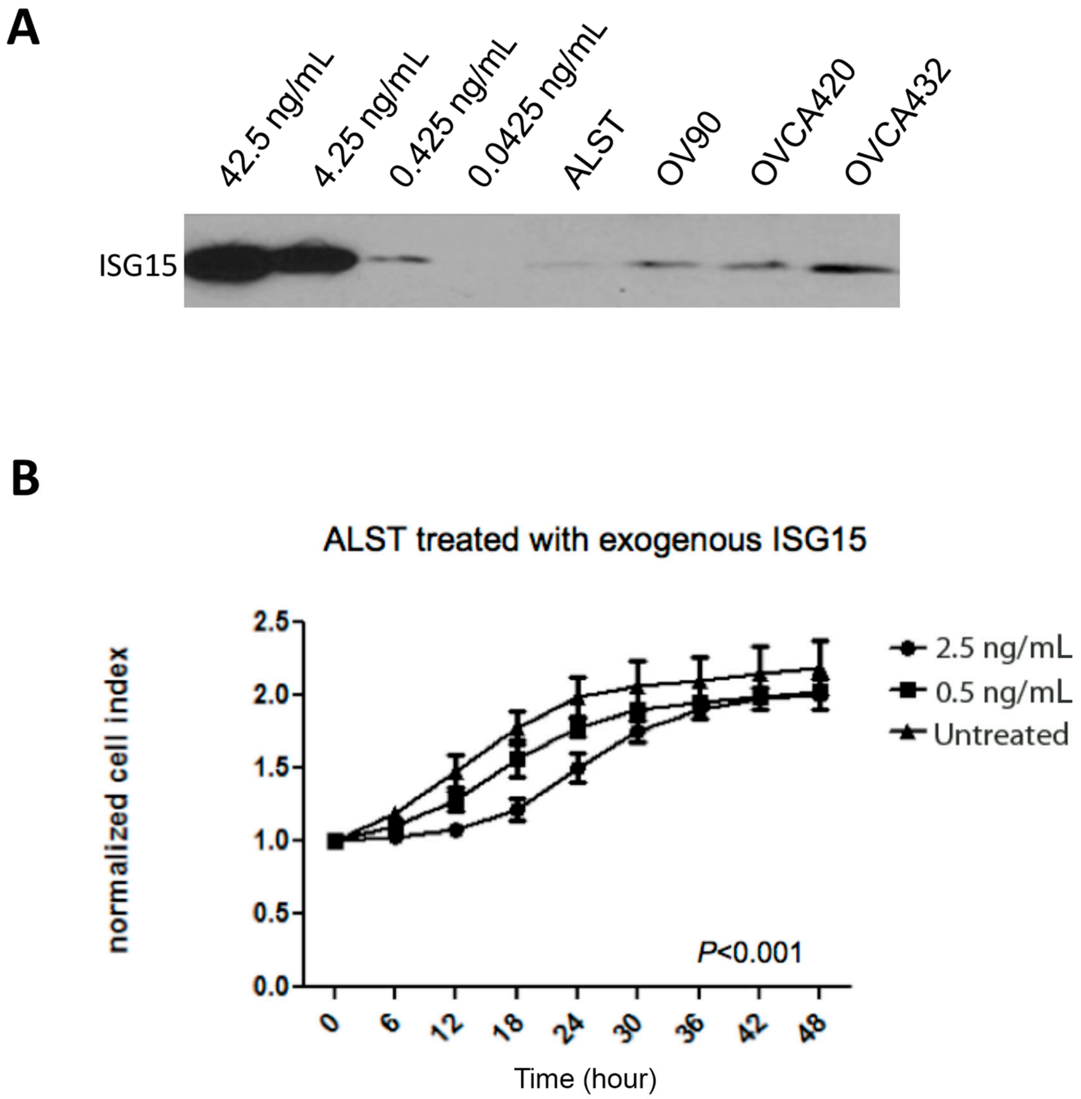

Figure S2. Exogenous ISG15 suppresses ovarian tumor growth in vitro. To evaluate whether ovarian cancer cells expressing high levels of endogenous ISG15 would secrete ISG15 protein into the extracellular space, ovarian cancer cell lines ALST, OV90, OVCA420, and OVCA432 were cultured in serum free media. (**A**) Western blot analyses on the conditioned media samples collected demonstrated a various amount of ISG15 in the conditioned media suggested secretion of ISG15 by cancer into the extracellular space. (**B**) To evaluate the effect of exogenous ISG15 on ovarian cancer growth, ALST cells were treated with 0.5 ng/mL or 2.5 ng/mL recombinant ISG15 protein. Proliferation assay results showed ISG15 treated ALST demonstrated a significant dosage dependent decrease in growth rate in the presence of exogenous ISG15 compared with that in PBS treated cells ($p < 0.001$).

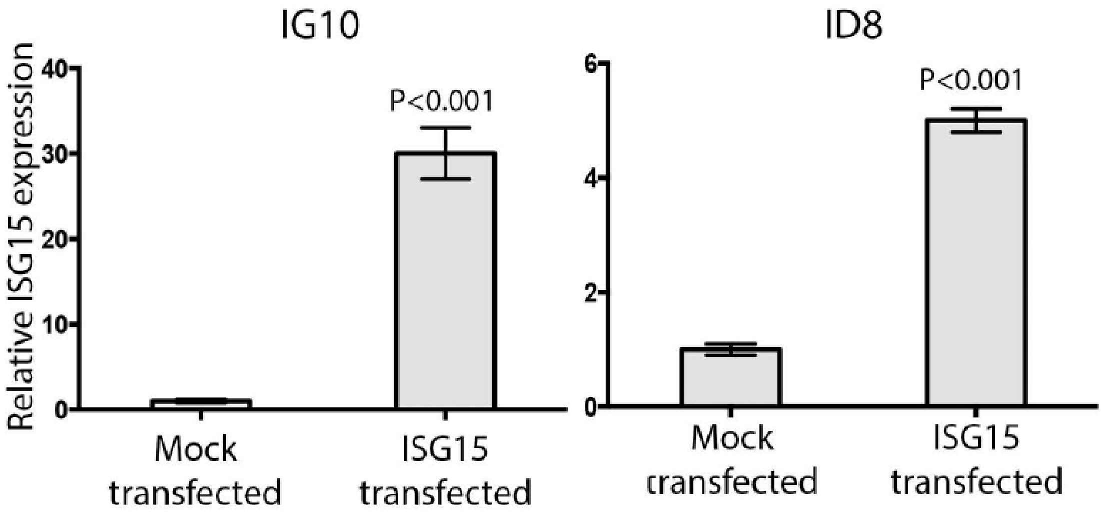

Figure S3. ISG15 overexpression in mouse ovarian cancer cell lines. Upregulation of ISG15 in ID8 and IG10 mouse ovarian cancer cell lines transfected with ISG15 overexpression construct was validated by quantitative real-time PCR.

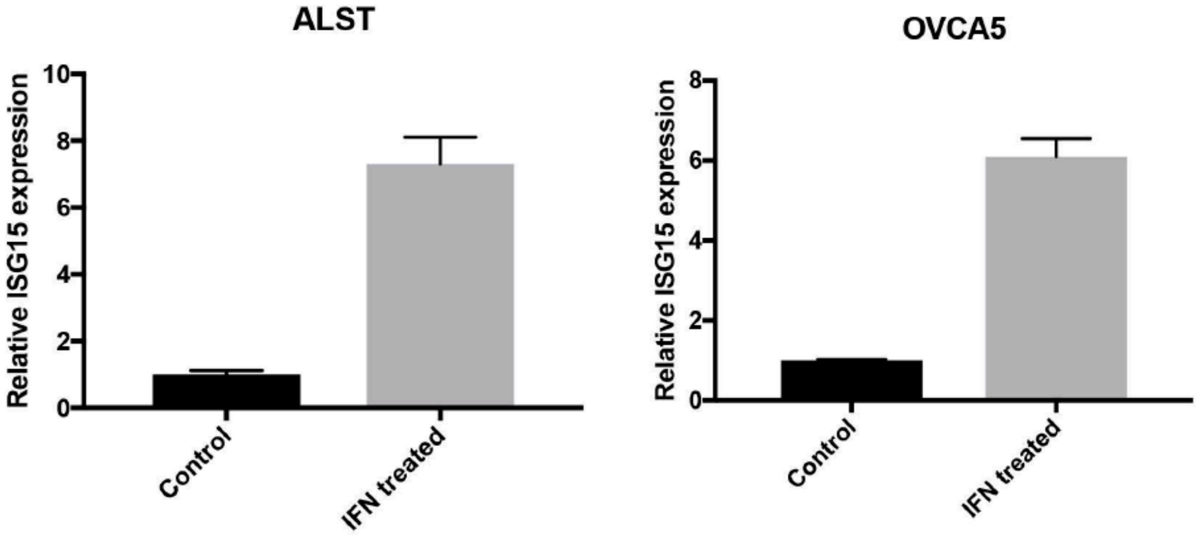

Figure S4. Interferon treatment on ovarian cancer cells upregulates ISG15 expression. Quantitative real-time PCR analysis on ALST and OVCA5 ovarian cancer cells treated with gamma interferon showed that interferon treatment significantly induced ISG15 expression in cancer cells when compared with treatment with control solvent.

Table S1. Multivariate survival analysis of ISG15 expression in ovarian cancer patients.

Overall Survival		
**Factor**	**Hazard Ratio (95% CI)**	***p*-Value**
Age	1.004 (0.987–1.022)	0.615
Debulking surgery	1.206 (0.707–2.057)	0.491
Low ISG15 expression	2.265 (1.498–3.425)	<0.001
**Progression-Free Survival**		
**Factor**	**Hazard Ratio (95% CI)**	***p*-Value**
Age	0.989 (0.974–1.004)	0.156
Debulking surgery	1.189 (0.698–2.025)	0.525
Low ISG15 expression	1.909 (1.284–2.837)	0.001

Table S2. Target proteins of ISGylation.

**Cell Motility**			
**Gene Symbol**	**Entrez Gene Name**	**Subcellular Localization**	**Protein Type**
ANXA2	Annexin A2	Plasma Membrane	other
DDX58	DEAD (Asp-Glu-Ala-Asp) box polypeptide 58	Cytoplasm	enzyme
ERK1	Extracellular Signal-Regulated Kinase 1	Cytoplasm	kinase
FLNA	Filamin A, alpha	Cytoplasm	other
HSPD1	Heat shock 60kDa protein 1 (chaperonin)	Cytoplasm	enzyme
LGMN	Legumain	Cytoplasm	peptidase
MSN	Moesin	Plasma Membrane	other
PLCG1	Phospholipase C, gamma 1	Cytoplasm	enzyme
STAT1	Signal transducer and activator of transcription 1, 91kDa	Nucleus	transcription regulator
VIL2	Ezrin	Plasma Membrane	other
VIM	Vimentin	Cytoplasm	other
**Cell Invasion**			
**Gene Symbol**	**Entrez Gene Name**	**Subcellular Localization**	**Protein Type**
GBP1	Guanylate binding protein 1, interferon-inducible	Cytoplasm	enzyme
GPI	Glucose-6-phosphate isomerase	Extracellular Space	enzyme
HSPCA	Heat shock protein 90kDa alpha (cytosolic), class A member 1	Cytoplasm	enzyme
MX1	Myxovirus (influenza virus) resistance 1, interferon-inducible protein p78 (mouse)	Cytoplasm	enzyme
PLCG1	Phospholipase C, gamma 1	Cytoplasm	enzyme
VIL2	Ezrin	Plasma Membrane	other
VIM	Vimentin	Cytoplasm	other
**Apoptosis**			
**Gene Symbol**	**Entrez Gene Name**	**Subcellular Localization**	**Protein Type**
ANXA5	Annexin A5	Plasma Membrane	other
CBX4	Chromobox homolog 4	Nucleus	transcription regulator
CFL1	Cofilin 1 (non-muscle)	Nucleus	other

CSNK2A1	Casein kinase 2, alpha 1 polypeptide	Cytoplasm	kinase
DDX58	DEAD (Asp-Glu-Ala-Asp) box polypeptide 58	Cytoplasm	enzyme
DNAJB1	DnaJ (Hsp40) homolog, subfamily B, member 4	Nucleus	other
EIF4G1	Eukaryotic translation initiation factor 4 gamma, 1	Cytoplasm	translation regulator
ELAVL1	ELAV (embryonic lethal, abnormal vision, Drosophila)-like 1 (Hu antigen R)	Cytoplasm	other
ERK1	Extracellular Signal-Regulated Kinase 1	Cytoplasm	kinase
GSTP1	Glutathione S-transferase pi 1	Cytoplasm	enzyme
HSPA1A	Heat shock 70kDa protein 1A	Cytoplasm	other
HSPA8	Heat shock 70kDa protein 8	Cytoplasm	enzyme
HSPCA	Heat shock protein 90kDa alpha (cytosolic), class A member 1	Cytoplasm	enzyme
HSPCB	Heat shock protein 90kDa alpha (cytosolic), class B member 1	Cytoplasm	enzyme
HSPD1	Heat shock 60kDa protein 1 (chaperonin)	Cytoplasm	enzyme
LGMN	Legumain	Cytoplasm	peptidase
MSN	Moesin	Plasma Membrane	other
MX1	Myxovirus (influenza virus) resistance 1, interferon-inducible protein p78 (mouse)	Cytoplasm	enzyme
PLCG1	Phospholipase C, gamma 1	Cytoplasm	enzyme
RBBP4	Retinoblastoma binding protein 4	Nucleus	enzyme
SENP1	SUMO1/sentrin specific peptidase 1	Nucleus	peptidase
STAT1	Signal transducer and activator of transcription 1, 91kDa	Nucleus	transcription regulator
TOP2A	Topoisomerase (DNA) II alpha 170kDa	Nucleus	enzyme
VCP	Valosin containing protein	Cytoplasm	enzyme
VIL2	Ezrin	Plasma Membrane	other
	**Cell Proliferation**		
**Gene Symbol**	**Entrez Gene Name**	**Subcellular Localization**	**Protein Type**
ANXA2	Annexin A2	Plasma Membrane	other
CSNK2A1	Casein kinase 2, alpha 1 polypeptide	Cytoplasm	kinase
DDB2	Damage-specific DNA binding protein 2, 48kDa	Nucleus	other
DNAJB1	DnaJ (Hsp40) homolog, subfamily B, member 4	Nucleus	other
DNAJB4	DnaJ (Hsp40) homolog, subfamily B, member 4	Nucleus	other
EIF4G1	Eukaryotic translation initiation factor 4 gamma, 1	Cytoplasm	translation regulator
ELAVL1	ELAV (embryonic lethal, abnormal vision, Drosophila)-like 1 (Hu antigen R)	Cytoplasm	other
ERK1	Extracellular Signal-Regulated Kinase 1	Cytoplasm	kinase
FLNA	Filamin A, alpha	Cytoplasm	other
GBP1	Guanylate binding protein 1, interferon-inducible	Cytoplasm	enzyme
GSTP1	Glutathione S-transferase pi 1	Cytoplasm	enzyme
HSPA1A	Heat shock 70kDa protein 1A	Cytoplasm	other
HSPD1	Heat shock 60kDa protein 1 (chaperonin)	Cytoplasm	enzyme
IFIT3	Interferon-induced protein with tetratricopeptide repeats 3	Cytoplasm	other
JAK1	Janus kinase 1	Cytoplasm	kinase
MX1	Myxovirus (influenza virus) resistance 1, interferon-inducible protein p78 (mouse)	Cytoplasm	enzyme

PFN1	Profilin 1	Cytoplasm	other
RBBP4	Retinoblastoma binding protein 4	Nucleus	enzyme
SENP1	SUMO1/sentrin specific peptidase 1	Nucleus	peptidase
STAT1	Signal transducer and activator of transcription 1, 91kDa	Nucleus	transcription regulator
TOP2A	Topoisomerase (DNA) II alpha 170kDa	Nucleus	enzyme
UBE2L6	Ubiquitin-conjugating enzyme E2L 6	Cytoplasm	enzyme
VIL2	Ezrin	Plasma Membrane	other

© 2018 by the authors. Licensee MDPI, Basel, Switzerland. This article is an open access article distributed under the terms and conditions of the Creative Commons Attribution (CC BY) license (http://creativecommons.org/licenses/by/4.0/).
